# Supplementary material for: AI-based identification of therapeutic agents targeting GPCRs: introducing ligand type classifiers and systems biology
Source: Chem Sci. 2023 Jul 24;14(32):8651–61. doi: 10.1039/d3sc02352d (PMC10430665; doi:10.1039/d3sc02352d)
Supplement: SC-014-D3SC02352D-s001 [file SC-014-D3SC02352D-s001.pdf]

Electronic Supplementary Material (ESI) for Chemical Science.
This journal is © The Royal Society of Chemistry 2023

MolPort SIA
Address: Lacplesa 41
Riga, LV-1011, Latvia
VAT No.: LV40003881547
www.molport.com

MolPort

PACKING LIST

549.

Packing list: LFF12R5049416

Customer ID: 11445

Issue date: Monday, August 6, 2018

Bill to:
Forschungszentrum Jülich
F-BK
52425 Julich
Germany
VAT no. DE122624631

Ship to:
Attn: W50/Elisabeth Bauer
Forschungszentrum Jülich
Warenannahme (M-ME) Gebaude 20.05
Polese 15.19 S179
Leo-Brandt-Strasse
52428 Julich
Germany
Phone: +49-2461613037

PO# 45216549

No	MolPort ID	Supplier	Catalogue no.	Quantity	Box ID
1	MolPort-000-686-300	Eximed	EiM07-00051	5 mg	FO01
2	MolPort-000-676-340	Otava, Ltd.	7017470408	5 mg	FO01
3	MolPort-000-677-254	Otava, Ltd.	7018710852	5 mg	FO01
4	MolPort-000-769-610	Pharmeks, Ltd.	PHAR101262	2 mg	FO01
5	MolPort-000-415-371	Vitas-M Laboratory, Ltd.	STK610353	5 mg	FO01
6	MolPort-000-464-571	Vitas-M Laboratory, Ltd.	STK816960	5 mg	FO01
7	MolPort-000-517-295	Vitas-M Laboratory, Ltd.	STL355030	5 mg	FO01
8	MolPort-000-437-073	Vitas-M Laboratory, Ltd. (Premium)	STK895192	5 mg	FO01
9	MolPort-001-005-831	Vitas-M Laboratory, Ltd. (Premium)	STK136010	5 mg	FO01
10	MolPort-001-020-836	Vitas-M Laboratory, Ltd. (Premium)	STK268019	5 mg	FO01
11	MolPort-001-498-328	Vitas-M Laboratory, Ltd. (Premium)	STK098454	5 mg	FO01
12	MolPort-001-598-049	Vitas-M Laboratory, Ltd. (Premium)	STK277079	5 mg	FO01

*This package contains 12 compounds shipped by TNT AWB# 607564845. Please review the contents of this shipment and contact sales@molport.com about any discrepancies within a week from receipt.

Wareneingang M-ME
Heimig, Tel. 5040
Lieferschein zurück an M-ME
07. Aug. 2018
sachlich richtig gekennzeichnet am:

Name in Klarschrift:	Unterschrift:

Pos. 1 - 12.
836 752

Contact us:
sales@molport.com
Tel: +371 67790398
Fax: +371 66117161
Skype: MolPort

Bank: Swedbank AS
Address: Balasta dambis 1a, Riga,
LV-1048, Latvija
IBAN: LV11HABA0551029918596
SWIFT: HABALV22

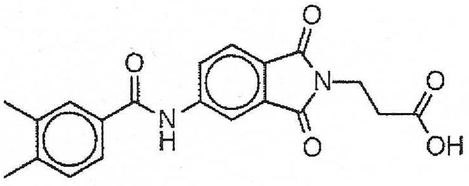

A2AR-JG-001
MolPort-001-598-049
O=C(O)CCN(C1=O)C(=O)c(c12)ccc(c2)NC(=O)c3cc(C)c(C)cc3
soluble

12

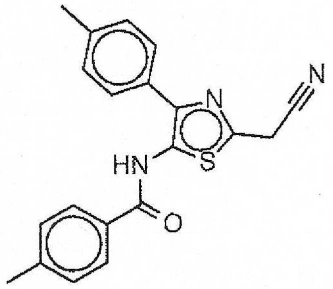

A2AR-JG-002
MolPort-000-464-571
Cc1ccc(cc1)-c2c(sc(n2)CC#N)NC(=O)c3ccc(C)cc3
soluble

6

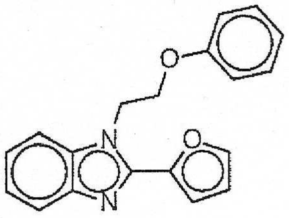

8

A2AR-JG-003
MolPort-000-437-073
o1cccc1-c(n2)n(c(c23)cccc3)CCOc4ccccc4
very

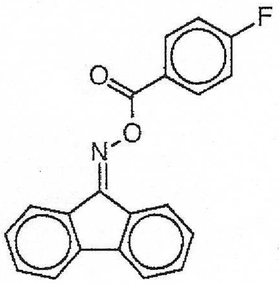

11

A2AR-JG-004
MolPort-001-498-328
c1cc(F)ccc1C(=O)ON=C(c2cccc3)c(c4c23)cccc4
poorly

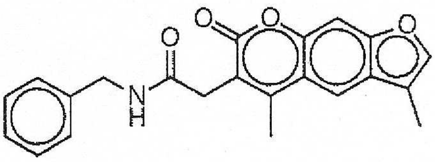

1

A2AR-JG-005
MolPort-000-686-300
c1ccccc1CNC(=O)Cc(c(=O)o2)c(C)c(c3)c2cc(c34)occ4C
moderately

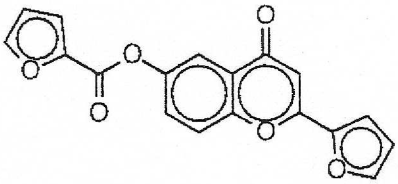

3

A2AR-JG-006
MolPort-000-677-254
o1cccc1C(=O)Oc(c2)ccc(c23)oc(cc3=O)-c4ccco4
soluble

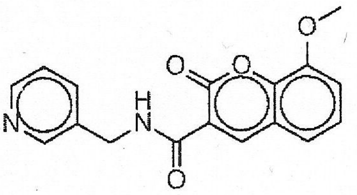

A2AR-JG-007
MolPort-000-517-295
c1ncccc1CNC(=O)c(c(=O)o2)cc(c23)cccc3OC
soluble

7

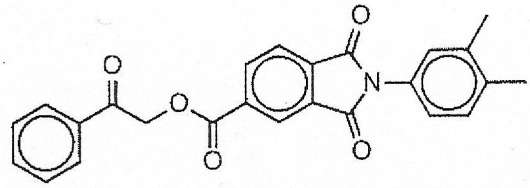

A2AR-JG-008
MolPort-001-020-836
c1ccccc1C(=O)COC(=O)c(c2)ccc(c23)C(=O)N
(C3=O)c(c4)ccc(C)c4C
moderately

10

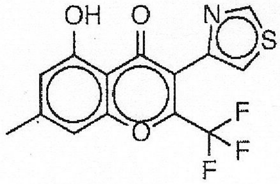

A2AR-JG-009
MolPort-000-769-610
n1cscc1-c(c2=O)c(C(F)(F)F)oc(c23)cc(C)cc3O
insoluble

4

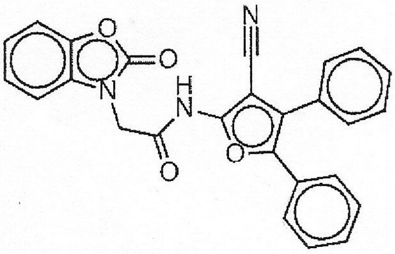

A2AR-JG-010
MolPort-001-005-831
c1ccccc1-c(c2C#N)c(-
c3ccccc3)oc2NC(=O)Cn4c(=O)oc(c45)cccc5
moderately

9

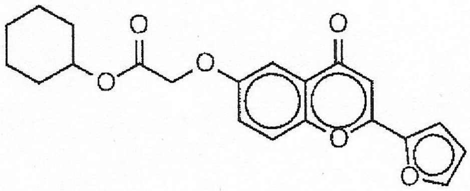

A2AR-JG-011
MolPort-000-676-340
C1CCCCC1OC(=O)COc(c2)ccc(c23)oc(cc3=O)-c4ccco4
very

2

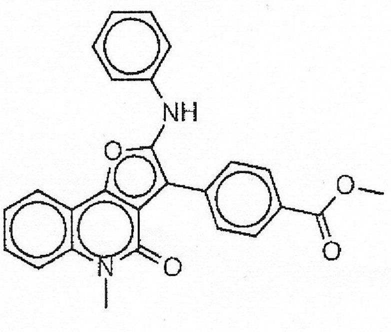

A2AR-JG-012
MolPort-000-415-371
COC(=O)c1ccc(cc1)-
c2c(Nc3ccccc3)oc(c24)c5c(n(C)c4=O)cccc5
moderately

5
