# Supplementary material for: AI-based identification of therapeutic agents targeting GPCRs: introducing ligand type classifiers and systems biology
Source: Chem Sci. 2023 Jul 24;14(32):8651–61. doi: 10.1039/d3sc02352d (PMC10430665; doi:10.1039/d3sc02352d)
Supplement: SC-014-D3SC02352D-s002 [file SC-014-D3SC02352D-s002.pdf]

Electronic Supplementary Material (ESI) for Chemical Science.
This journal is © The Royal Society of Chemistry 2023

Supporting Information for

AI-based identification of therapeutic agents targeting GPCRs: introducing ligand type classifiers and systems biology

Jonas Goßen,[1,2†] Rui Pedro Ribeiro,[1†] Dirk Bier,[3] Bernd Neumaier,[3] Paolo Carloni,[1,4] Alejandro Giorgetti,[1,5] Giulia Rossetti[1,6,7*]

[1]Institute for Computational Biomedicine (INM-9 / IAS-5) Forschungszentrum Jülich
[2]Faculty of Mathematics, Computer Science and Natural Sciences RWTH Aachen University
[3]Institut für Neurowissenschaften und Medizin INM-5: Nuklearchemie Forschungszentrum Jülich
[4]JARA-Institut Molecular Neuroscience and Neuroimaging (INM-11) Forschungszentrum Jülich
[5]Department of Biotechnology University of Verona
[6]Jülich Supercomputing Centre (JSC) Forschungszentrum Jülich
[7]Department of Neurology University Hospital Aachen (UKA), RWTH Aachen University

*Giulia Rossetti.
Email: g.rossetti@fz-juelich.de

This PDF file includes:

Figures S1 to S8
Tables S1 to S7
Filtering Criteria for Agonist/Antagonist Data

Fig. S1.

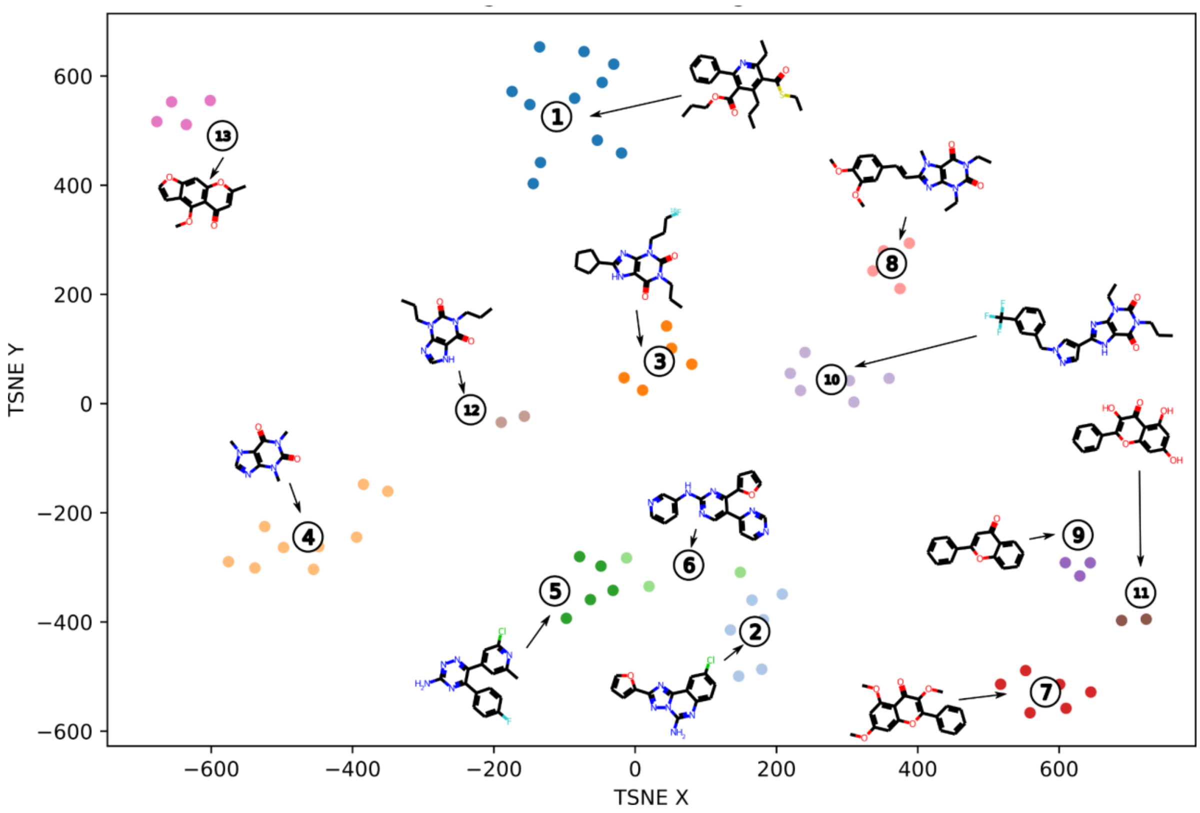

TSNE representation of considered antagonists coming from GtoP, DrugBank and GPCR DB clustered with hierarchical clustering of 13 clusters. Outliers were removed by hand and selected cluster representatives are shown. The chemical space of antagonists is more diverse than that of agonists.

Fig. S2.

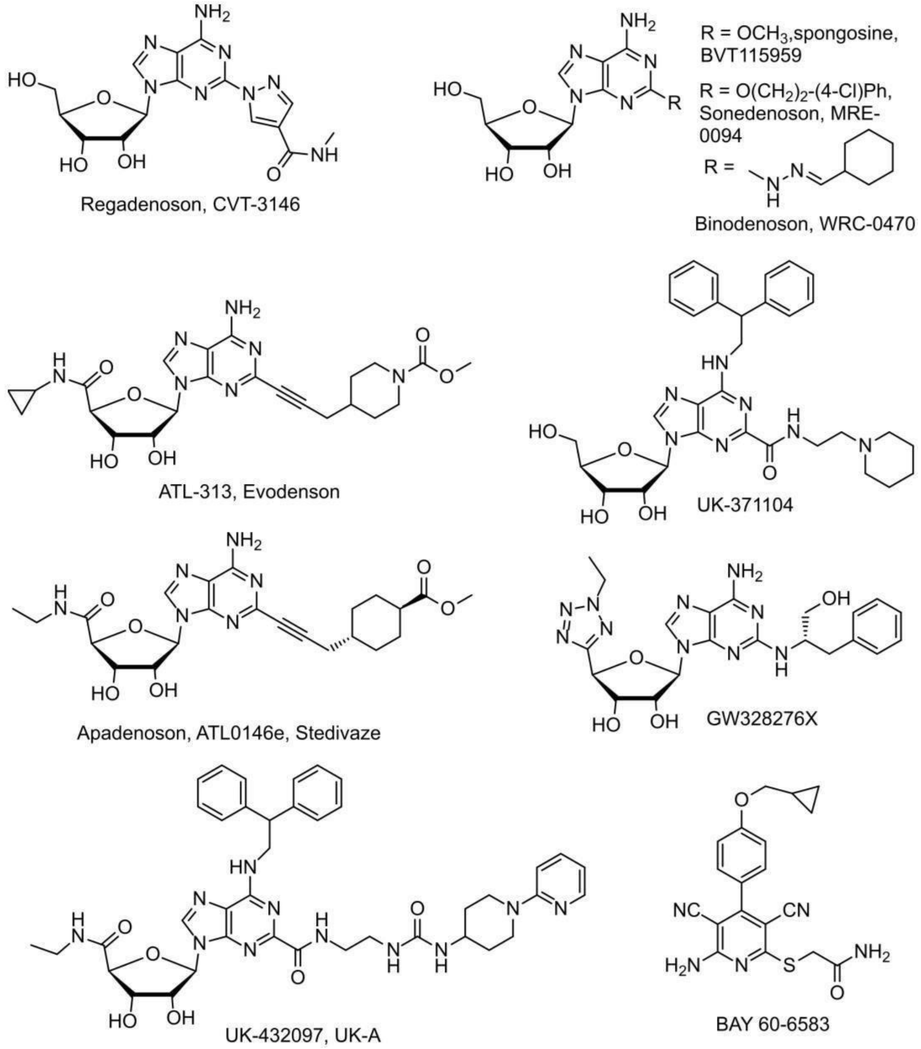

Most of the selective agonists for A2A contain a ribose and an adenine-like moiety.

Fig. S3.

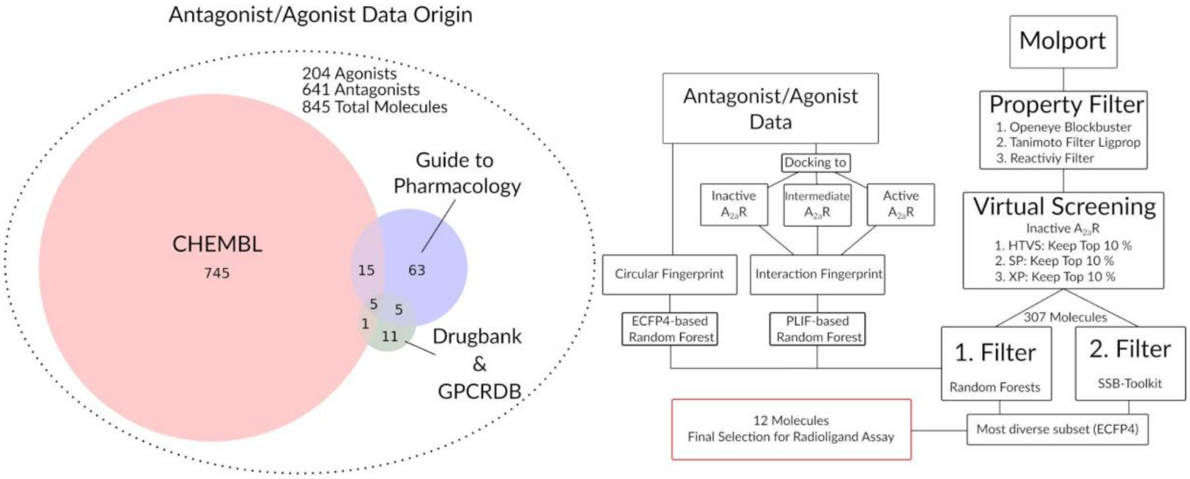

Overview of data collection (left) and overall workflow overview (right).

Fig. S4.

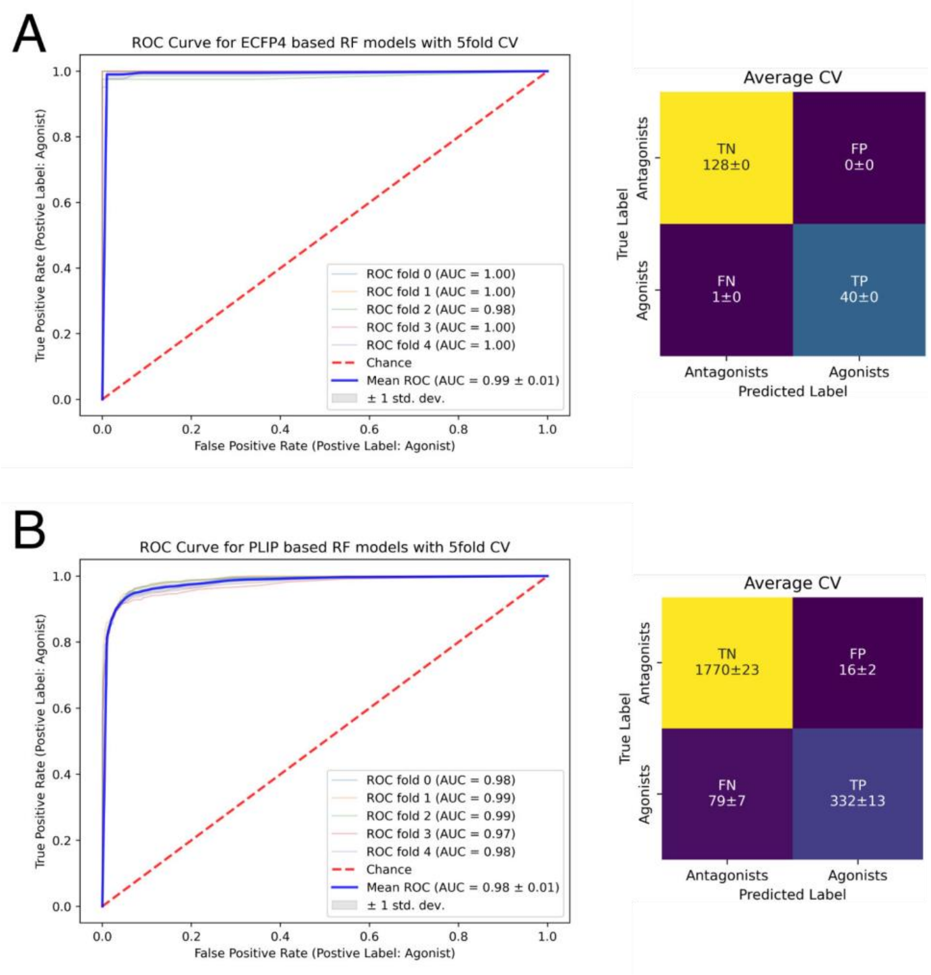

Confusion matrix and ROC plot of (A) ECFP4 based and (B) PLIF based random forest model. Validated by 5-fold-cross validation. In the PLIF Based Random Forest model, the best 5 poses for each unique ligand were considered for the classifier. Therefore, we applied group cross-validation that ensures that no molecule is shared in between training and test splits. For the ECFP4 model every unique molecule in the training sets was used. The classification by the docking-based approach shows a high accuracy score for the respective splits (compare Table SI 3). Although the area under the curve shows a high value of 0.98, a bit lower than our model based upon the chemical ECFP4 fingerprint, the accuracy of both models is still comparable.

Fig. S5.

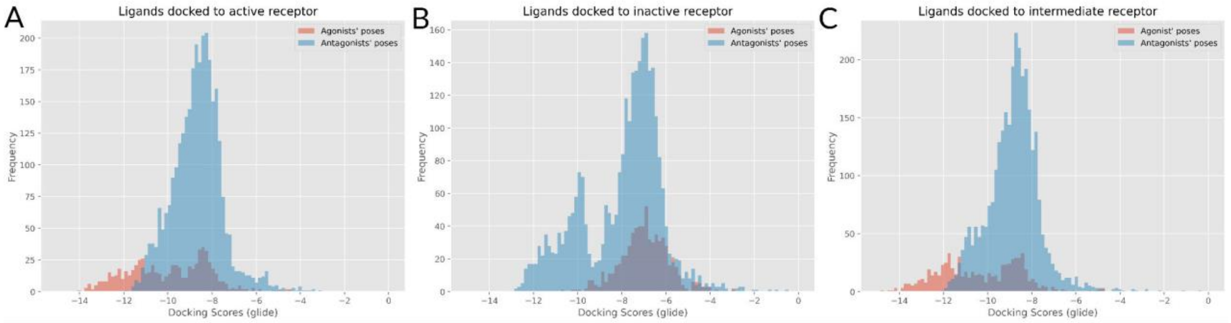

Glide docking scores of the assembled agonist/antagonist library on the three receptors in active(5G53)//inactive(4EIY58)/intermediate(2YDO) conformation. The average agonist binds with a higher score to the A2A in the active state compared to the inactive state.

Fig. S6.

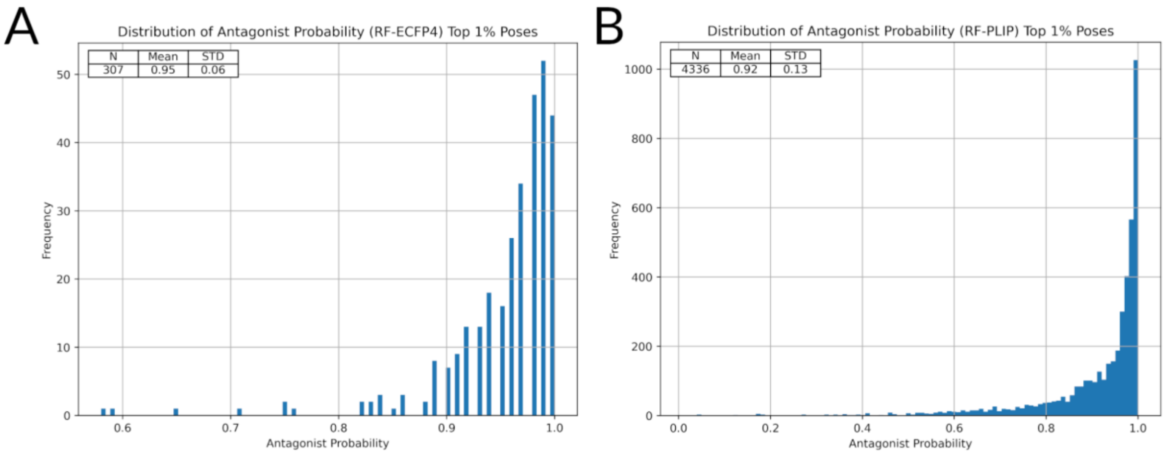

Estimated antagonist probability histograms (100 bins) of top 1% molecules selected by virtual screening to the A2AR. A: ECFP4-based RF classifier B: PLIF-based RF classifier. Number of samples (N), arithmetic mean (Mean) and standard deviation (STD) are given for both distributions.

Fig. S7.

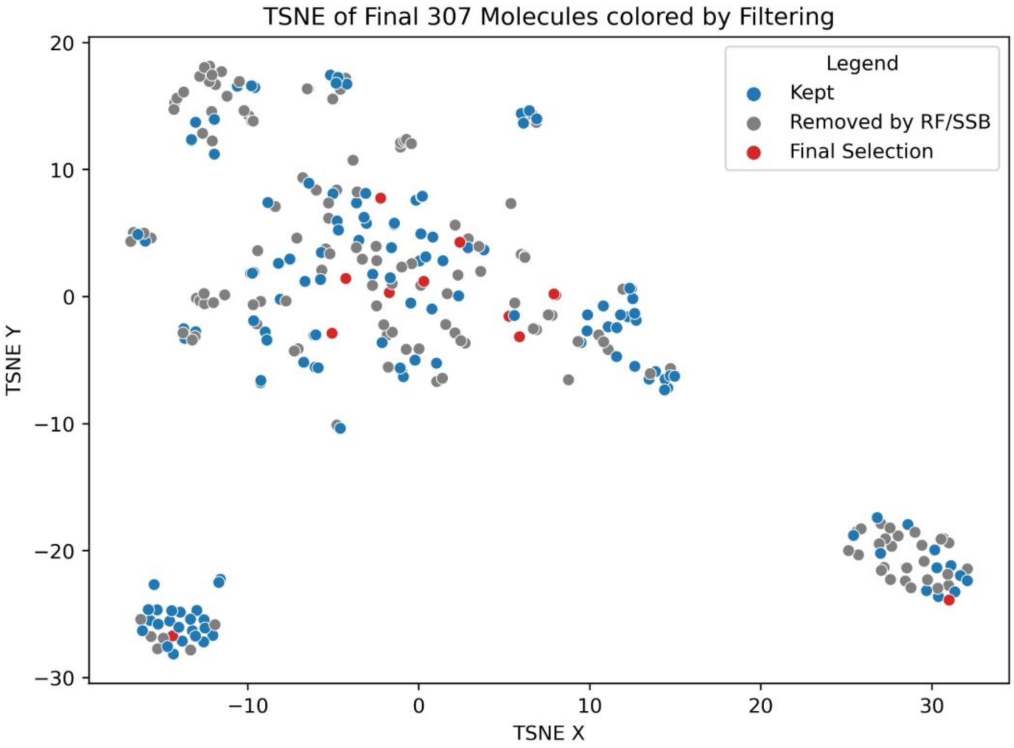

TSNE representation of the 307 molecules identified through virtual screening. Tanimoto similarity was used for the generation of the distance matrix used by the TSNE algorithm. PCA with 50 dimensions was applied.

Fig. S8.

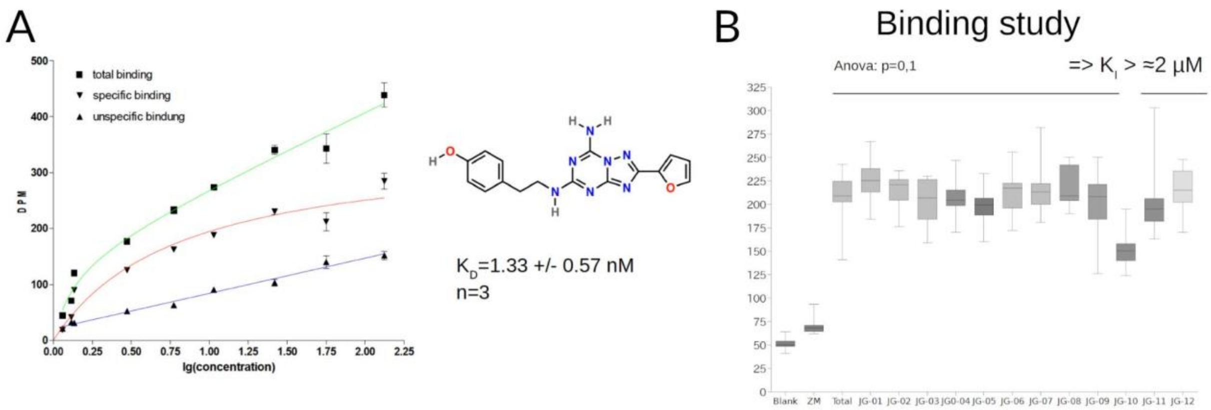

Calibration of assay to gold standard ZMA. (B) Binding study of final 12 selected molecules.

Table S1.

Method	PDB	Res. (Å)	State	Degree active (%)	Name	Function	PDB Date	Ref.
X-ray	**8CU6**	2.8	Inactive	1	LJ-4517	Antagonist	31/08/2022	1
X-ray	8CU7	2.1	Inactive	1	LJ-4517	Antagonist	31/08/2022	1
X-ray	**8DU3**	2.5	Inactive	1	21a	Antagonist	10/08/2022	2
cryo-EM	7T32	3.4	Inactive	1	ZM-241385	Antagonist	10/08/2022	3
X-ray	**7EZC**	3.8	Active	73	UK-432,097	Agonist	13/04/2022	4
X-ray	7PYR	2.6	Inactive	1	Preladenant conjugate PSB-2115	Antagonist	02/03/2022	5
X-ray	**7PX4**	2.3	Inactive	1	Preladenant conjugate PSB-2113	Antagonist	02/03/2022	5
MicroED	7RM5	2.8	Inactive	1	ZM-241385	Antagonist	08/09/2021	6
X-ray	**7ARO**	3.1	Inactive	1	ChEMBL124 345	Agonist (partial)	07/04/2021	7
X-ray	6LPK	1.8	Inactive	1	ZM-241385	Antagonist	25/11/2020	8
X-ray	**6LPL**	2.0	Inactive	1	ZM-241385	Antagonist	25/11/2020	8
X-ray	6LPJ	1.8	Inactive	1	ZM-241385	Antagonist	25/11/2020	8

X-ray	**6WQA**	2.0	Inactive	1	ZM-241385	Antagonist	18/11/2020	9
X-ray	6ZDV	2.1	Inactive	1	2-Methyl-3-(4-methylthiazol-2-yl)-4-oxo-6-propyl-4H-chromen-7-yl acetate	Antagonist	16/09/2020	10
X-ray	**6ZDR**	1.9	Inactive	1	ChEMBL 2030687	Antagonist	16/09/2020	10
X-ray	6S0Q	2.7	Inactive	1	ZM-241385	Antagonist	15/07/2020	11
X-ray	**6S0L**	2.7	Inactive	1	ZM-241385	Antagonist	15/07/2020	11
X-ray	6PS7	1.9	Inactive	1	ZM-241385	Antagonist	13/11/2019	12
X-ray	**6JZH**	2.3	Inactive	1	ZM-241385	Antagonist	30/10/2019	13
X-ray	6GT3	2.0	Inactive	1	imaradenant	Antagonist	26/06/2019	14
X-ray	**6MH8**	4.2	Inactive	1	ZM-241385	Antagonist	24/04/2019	15
cryo-EM	6GDG	4.1	Active	100	NECA	Agonist	16/05/2018	16
X-ray	**5WF6**	2.9	Active	73	UK-432,097	Agonist	21/02/2018	17
X-ray	5WF5	2.6	Active	73	UK-432,097	Agonist	21/02/2018	17
X-ray	**5OLV**	2.0	Inactive	1	ChEMBL 1671936	Antagonist	17/01/2018	18
X-ray	5OLO	3.1	Inactive	1	tozadenant	Antagonist	17/01/2018	18

X-ray	**5OM4**	2.0	Inactive	1	ChEMBL 2024114	Antagonist	17/01/2018	18
X-ray	5OLG	1.9	Inactive	1	ZM-241385	Antagonist	17/01/2018	18
X-ray	**5OLZ**	1.9	Inactive	1	ChEMBL 2024114	Antagonist	17/01/2018	18
X-ray	5OLH	2.6	Inactive	1	vipadenant	Antagonist	17/01/2018	18
X-ray	**5OM1**	2.1	Inactive	1	ChEMBL 2024114	Antagonist	17/01/2018	18
X-ray	6AQF	2.5	Inactive	1	ZM-241385	Antagonist	10/01/2018	19
X-ray	**5VRA**	2.4	Inactive	1	ZM-241385	Antagonist	13/12/2017	20
X-ray	5NM2	2.0	Inactive	1	ZM-241385	Antagonist	27/09/2017	21
X-ray	**5NM4**	1.7	Inactive	1	ZM-241385	Antagonist	27/09/2017	21
X-ray	5NLX	2.1	Inactive	1	ZM-241385	Antagonist	27/09/2017	21
X-ray	**5MZJ**	2.0	Inactive	1	theophylline	Antagonist	26/07/2017	22
X-ray	5MZP	2.1	Inactive	1	caffeine	Antagonist	26/07/2017	22
X-ray	**5N2R**	2.8	Inactive	1	PSB36	Antagonist	26/07/2017	22
X-ray	5JTB	2.8	Inactive	1	ZM-241385	Antagonist	31/05/2017	23
X-ray	**5UVI**	3.2	Inactive	1	ZM-241385	Antagonist	24/05/2017	24

X-ray	5UIG	3.5	Inactive	16	5-Amino-N-[(2-Methoxyphenyl)methyl]-2-(3-Methylphenyl)-2h-1,2,3-Triazole-4-Carboximidamide	Antagonist	08/02/2017	25
X-ray	**5K2C**	1.9	Inactive	1	ZM-241385	Antagonist	21/09/2016	26
X-ray	5K2A	2.5	Inactive	1	ZM-241385	Antagonist	21/09/2016	26
X-ray	**5K2B**	2.5	Inactive	1	ZM-241385	Antagonist	21/09/2016	26
X-ray	5K2D	1.9	Inactive	1	ZM-241385	Antagonist	21/09/2016	26
X-ray	**5G53**	3.4	Active	100	NECA	Agonist	03/08/2016	27
X-ray	5IUA	2.2	Inactive	1	2-(Furan-2-yl)-5-N-[3-(4-phenylpiperazin-1-yl)propyl]-1H-[1,2,4]triazolo[1,5-a][1,3,5]triazin-8-ium-5,7-diamine	Antagonist	29/06/2016	28
X-ray	**5IU7**	1.9	Inactive	1	2-(Furan-2-yl)-5-N-[2-(4-phenylpiperidin-1-yl)ethyl]-1H-[1,2,4]triazolo[1,5-a][1,3,5]triazin-8-ium-5,7-diamine	Antagonist	29/06/2016	28
X-ray	5IU8	2.0	Inactive	1	ChEMBL 3934661	Antagonist	29/06/2016	28

X-ray	**5IU4**	1.7	Inactive	1	ZM-241385	Antagonist	29/06/2016	28
X-ray	5IUB	2.1	Inactive	1	ChEMBL 184061	Antagonist	29/06/2016	28
X-ray	**4UG2**	2.6	Active	73	CGS 21680	Agonist	08/04/2015	29
X-ray	4UHR	2.6	Active	73	CGS 21680	Agonist	08/04/2015	29
X-ray	**4EIY**	1.8	Inactive	1	ZM-241385	Antagonist	25/07/2012	30
X-ray	3UZC	3.3	Inactive	1	ChEMBL 2024114	Antagonist	21/03/2012	31
X-ray	**3UZA**	3.3	Inactive	1	compound 4g [PMID: 22220592]	Antagonist	21/03/2012	31
X-ray	3VG9	2.7	Inactive	1	ZM-241385	Antagonist	01/02/2012	32
X-ray	**3VGA**	3.1	Inactive	1	ZM-241385	Antagonist	01/02/2012	32
X-ray	3PWH	3.3	Inactive	1	ZM-241385	Antagonist	07/09/2011	33
X-ray	**3REY**	3.3	Inactive	1	xanthine amine congener	Antagonist	07/09/2011	33
X-ray	3RFM	3.6	Inactive	1	caffeine	Antagonist	07/09/2011	33
X-ray	**2YDO**	3.0	Active	73	adenosine	Agonist	18/05/2011	34
X-ray	2YDV	2.6	Active	73	NECA	Agonist	18/05/2011	34

X-ray	**3QAK**	2.7	Active	73	UK-432,097	Agonist	09/03/2011	35
X-ray	**3EML**	2.6	Inactive	1	ZM-241385	Antagonist	14/10/2008	36

Structural $A_{2a}R$ Data by Experiment.

Table S2.

PLIF Based RF Classifier Performance Evaluation Metrics

#CV	test_roc_auc	test_accuracy	test_precision	test_recall	test_f1
1	0.98	0.96	0.97	0.82	0.89
2	0.98	0.95	0.97	0.76	0.85
3	0.99	0.97	0.94	0.88	0.90
4	0.99	0.96	0.97	0.83	0.90
5	0.97	0.95	0.94	0.79	0.86
**Average**	0.98±0.01	0.96±0.01	0.96 ± 0.01	0.82±0.02	0.88±0.01

ECFP4 Based RF Classifier Performance Evaluation Metrics

#CV	test_roc_auc	test_accuracy	test_precision	test_recall	test_f1
1	1.0	1.0	1.0	1.0	1.0
2	1.0	1.0	1.0	1.0	1.0
3	1.0	1.0	1.0	1.0	1.0
4	0.98	0.97	1.00	0.88	0.94
5	0.97	0.98	1.00	0.93	0.96

PLIF Based RF Classifier Performance Evaluation Metrics

#CV	test_roc_auc	test_accuracy	test_precision	test_recall	test_f1
1	0.98	0.96	0.97	0.82	0.89
2	0.98	0.95	0.97	0.76	0.85
3	0.99	0.97	0.94	0.88	0.90
4	0.99	0.96	0.97	0.83	0.90
5	0.97	0.95	0.94	0.79	0.86
**Average**	0.98±0.01	0.96±0.01	0.96 ± 0.01	0.82±0.02	0.88±0.01

ECFP4 Based RF Classifier Performance Evaluation Metrics

#CV	test_roc_auc	test_accuracy	test_precision	test_recall	test_f1
**Average**	0.99±0.01	0.99±0.01	1±0	0.96±0.03	0.98±0.01

Selected performance evaluation metrics generated with 5-fold cross-validation. CV types are StratifiedGroupKFold (PLIF) and StratifiedKFold (ECFP4).

Table S3.

ID	ECFP4-RF Antagonist Probability	PLIF-RF Antagonist Probability
JG-001	0.92	0.82
JG-002	1.00	0.82
JG-003	1.00	0.82
JG-004	0.90	0.82
JG-005	0.94	0.82
JG-006	0.99	0.82
JG-007	1.00	0.82
JG-008	1.00	0.82
JG-009	1.00	0.82
JG-010	0.98	0.82
JG-011	0.96	0.82

Predicted probability for the 12 selected compounds to be antagonists according to ECFP4 and PLIF based random forest classifiers.

Table S4.

*values obtained with Kdeep trained against the PDBbind v.2016[37]

Ligand Alias	pKd*	Ki (μM)	IC50 (μM)	pIC50
JG-01	6.5	4.0	0.1	7.1
JG-02	6.2	6.6	0.1	6.9
JG-03	5.1	94.2	1.8	5.7
JG-04	6.0	11.5	0.2	6.6
JG-05	6.0	11.3	0.2	6.7
JG-06	5.5	35.0	0.7	6.2
JG-07	5.8	16.8	0.3	6.5
JG-08	6.0	11.3	0.2	6.7
JG-09	5.7	24.8	0.5	6.3
JG-10	6.8	1.7	0.0	7.5
JG-11	5.7	25.2	0.5	6.3

Results obtained from our SB approach applied to the best binding poses of the 12 most promising molecules from the virtual screening towards the A2A receptor.

Table S5.

Title	IFDScore XP	GScore
JG-01	-584.37	-13.119
JG-02	-582.49	-12.961
JG-03	-579	-11.454
JG-04	-580.23	-12.589
JG-05	-582.37	-12.576
JG-06	-585.58	-12.314
JG-07	-581.77	-11.601
JG-08	-582.34	-14.172
JG-09	-588.98	-11.241
JG-10	-585.45	-14.55
JG-11	-587.62	-13.468
JG-12	-584.22	-14.474

Induced Fit redocking of compounds selected by virtual screening workflow.

Table S6.

Hydrogen Bonds

Index	Residue	Distance H-A	Distance D-A	Donor Angle	Donor Atom	Acceptor Atom
1	PHE168	2.8	3.2	104.93	1246	2352 [O2]
2	GLU169	2.18	3.17	175.61	1257	2352 [O2]
3	ASN253	1.94	2.8	144.47	1863	2336 [N1]

Pi-Stacking

Index	Residue	Distance	Angle	Offset	Ligand Atoms
1	PHE168	3.57	8.04	0.74	2333, 2334, 2337, 2344, 2345

Hydrophobic Interactions

Index	Residue	Distance	Ligand Atom	Protein Atom
1	ALA63	3.6	2340	473
2	ILE66	3.74	2356	495
3	VAL84	3.48	2341	621
4	VAL84	3.54	2327	621
5	LEU85	3.6	2328	629
6	PHE168	3.88	2332	1256
7	LEU249	3.32	2331	1829
8	LEU267	3.96	2359	1971

9	TYR271	3.78	2357	2011
10	TYR271	3.57	2358	2009
11	ILE274	3.71	2343	2033
12	ILE274	3.81	2359	2035

Summary of polar and non-polar contacts of JG-10 in the induced-fit pose.

Table S7.

ID	T_c	Molecule	Most-similar in ChEMBL	ID	T_c	Molecule	Most-similar in ChEMBL
[1]	0.35	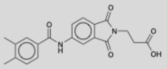	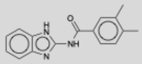	[7]	0.67	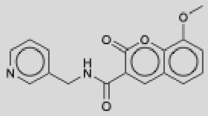	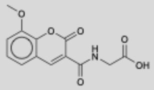
[2]	0.47	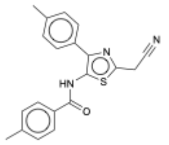	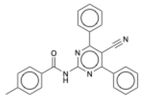	[8]	0.31	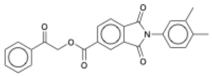	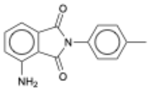
[3]	0.48	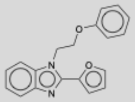	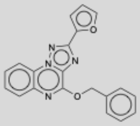	[9]	0.35	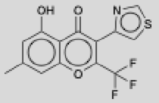	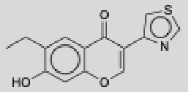
[4]	0.27	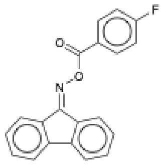	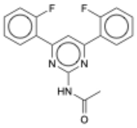	[10]	0.33	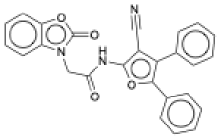	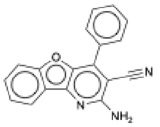
[5]	0.37	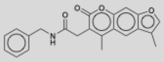	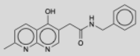	[11]	0.33	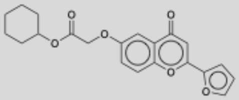	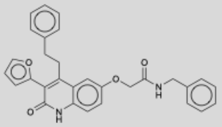
[6]	0.35	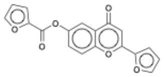	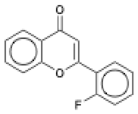	[12]	0.36	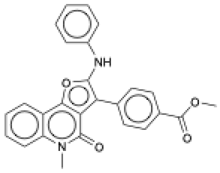	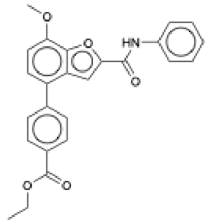

Tanimoto Similarity Score of candidates selected for experimental testing comparison to most similar known A2A binders found in ChEMBL database [38]

.

Filtering Criteria for Agonist/Antagonist Data

For the extraction and filtering we used the following criteria for the databases:

1. ChEMBL. Version: ChEMBL29 (Downloaded July 2021)
   Filtering as follows for finding agonists. Excluded molecules according to these criteria were called antagonists.

a. assay_type: “F” (Functional)
b. standard_relation: “=”
c. Value: >0
d. Assay description contains: ‘Agonist activity’
e. Assay description does not contain any of the following substrings: 'Antagonist activity','Antagonistic activity', 'Antagonist potency', 'Inverse agonist','Inhibition of human','Relaxant activity','Inhibition of the effects of NECA','Inhibition of 1','Binding affinity to human'

2. DrugBank. Target Adenosine receptor A_{2A}, action criterium “agonist, antagonist or inhibitor” (Downloaded on 02.08.2021)
3. GuideToPharmacology. Target: A_{2A} receptor (Agonist and Antagonist Tables) (Downloaded on 03.08.2021)

GPCRDB. Target: A_{2a}R, Mechanism of Action: Agonist/Antagonist, (Downloaded on 03.08.2021)

Duplicates were removed with Schrödingers “unique_smiles” utility program.

References

1 A. Shiriaeva, D. Park, G. Kim, Y. Lee, X. Hou, D. B. Jarhad, G. Kim, J. Yu, Y. E. Hyun, W. Kim, Z.-G. Gao, K. A. Jacobson, G. W. Han, R. C. Stevens, L. S. Jeong, S. Choi and V. Cherezov, *J. Med. Chem.*, , DOI:10.1021/acs.jmedchem.2c00462.

2 R. Bolteau, R. Duroux, A. Laversin, B. Vreulz, A. Shiriaeva, B. Stauch, G. W. Han, V. Cherezov, N. Renault, A. Barczyk, S. Ravez, M. Coevoet, P. Melnyk, M. Liberelle and S. Yous, *European Journal of Medicinal Chemistry*, 2022, **241**, 114620.

3 K. Zhang, H. Wu, N. Hoppe, A. Manglik and Y. Cheng, *Nat Commun*, 2022, **13**, 4366.

4 M. Cui, Q. Zhou, Y. Xu, Y. Weng, D. Yao, S. Zhao and G. Song, *IUCrJ*, 2022, **9**, 333–341.

5 T. Claff, T. A. Klapschinski, U. K. Tiruttani Subhramanyam, V. J. Vaaßen, J. G. Schlegel, C. Vielmuth, J. H. Voß, J. Labahn and C. E. Müller, *Angew Chem Int Ed Engl*, 2022, **61**, e202115545.

6 M. W. Martynowycz, A. Shiriaeva, X. Ge, J. Hattne, B. L. Nannenga, V. Cherezov and T. Gonen, *Proc Natl Acad Sci U S A*, 2021, **118**, e2106041118.

7 T. Amelia, J. P. D. van Veldhoven, M. Falsini, R. Liu, L. H. Heitman, G. J. P. van Westen, E. Segala, G. Verdon, R. K. Y. Cheng, R. M. Cooke, D. van der Es and A. P. IJzerman, *J Med Chem*, 2021, **64**, 3827–3842.

8 K. Ihara, M. Hato, T. Nakane, K. Yamashita, T. Kimura-Someya, T. Hosaka, Y. Ishizuka-Katsura, R. Tanaka, T. Tanaka, M. Sugahara, and others, *Scientific reports*, 2020, **10**, 1–12.

9 M.-Y. Lee, J. Geiger, A. Ishchenko, G. W. Han, A. Barty, T. A. White, C. Gati, A. Batyuk, M. S. Hunter, A. Aquila, and others, *IUCrJ*.

10 W. Jespers, G. Verdon, J. Azuaje, M. Majellaro, H. Keränen, X. García-Mera, M. Congreve, F. Deflorian, C. de Graaf, A. Zhukov, and others, *Angewandte Chemie International Edition*, 2020, **59**, 16536–16543.

11 K. Nass, R. Cheng, L. Vera, A. Mozzanica, S. Redford, D. Ozerov, S. Basu, D. James, G. Knopp, C. Cirelli, and others, *IUCrJ*.

12 A. Ishchenko, B. Stauch, G. W. Han, A. Batyuk, A. Shiriaeva, C. Li, N. Zatsepin, U. Weierstall, W. Liu, E. Nango, and others, *IUCrJ*, 2019, **6**, 1106–1119.

13 Y. Shimazu, K. Tono, T. Tanaka, Y. Yamanaka, T. Nakane, C. Mori, K. Terakado Kimura, T. Fujiwara, M. Sugahara, R. Tanaka, and others, *Journal of applied crystallography*, 2019, **52**, 1280–1288.

14 A. Borodovsky, C. M. Barbon, Y. Wang, M. Ye, L. Prickett, D. Chandra, J. Shaw, N. Deng, K. Sachsenmeier, J. D. Clarke, and others, *Journal for immunotherapy of cancer*.

15 J. M. Martin-Garcia, L. Zhu, D. Mendez, M.-Y. Lee, E. Chun, C. Li, H. Hu, G. Subramanian, D. Kissick, C. Ogata, and others, *IUCrJ*, 2019, **6**, 412–425.

16 J. García-Nafría, Y. Lee, X. Bai, B. Carpenter and C. G. Tate, *Elife*, 2018, **7**, e35946.

17 K. L. White, M. T. Eddy, Z.-G. Gao, G. W. Han, T. Lian, A. Deary, N. Patel, K. A. Jacobson, V. Katritch and R. C. Stevens, *Structure*, 2018, **26**, 259–269.

18 P. Rucktooa, R. K. Cheng, E. Segala, T. Geng, J. C. Errey, G. A. Brown, R. M. Cooke, F. H. Marshall and A. S. Doré, *Scientific reports*, 2018, **8**, 1–7.

19 M. T. Eddy, M.-Y. Lee, Z.-G. Gao, K. L. White, T. Didenko, R. Horst, M. Audet, P. Stanczak, K. M. McClary, G. W. Han, and others, *Cell*, 2018, **172**, 68–80.

20 J. Broecker, T. Morizumi, W.-L. Ou, V. Klingel, A. Kuo, D. J. Kissick, A. Ishchenko, M.-Y. Lee, S. Xu, O. Makarov, and others, *Nature protocols*, 2018, **13**, 260–292.

21 T. Weinert, N. Olieric, R. Cheng, S. Brünle, D. James, D. Ozerov, D. Gashi, L. Vera, M. Marsh, K. Jaeger, and others, *Nature communications*, 2017, **8**, 1–11.

22 R. K. Cheng, E. Segala, N. Robertson, F. Deflorian, A. S. Doré, J. C. Errey, C. Fiez-Vandal, F. H. Marshall and R. M. Cooke, *Structure*, 2017, **25**, 1275–1285.

23 I. Melnikov, V. Polovinkin, K. Kovalev, I. Gushchin, M. Shevtsov, V. Shevchenko, A. Mishin, A. Alekseev, F. Rodriguez-Valera, V. Borshchevskiy, and others, *Science advances*, 2017, **3**, e1602952.

24 J. M. Martin-Garcia, C. E. Conrad, G. Nelson, N. Stander, N. A. Zatsepin, J. Zook, L. Zhu, J. Geiger, E. Chun, D. Kissick, and others, *IUCrJ*, 2017, **4**, 439–454.

25 B. Sun, P. Bachhawat, M. L.-H. Chu, M. Wood, T. Ceska, Z. A. Sands, J. Mercier, F. Lebon, T. S. Kobilka and B. K. Kobilka, *Proceedings of the National Academy of Sciences*, 2017, **114**, 2066–2071.
26 A. Batyuk, L. Galli, A. Ishchenko, G. W. Han, C. Gati, P. A. Popov, M.-Y. Lee, B. Stauch, T. A. White, A. Barty, and others, *Science advances*, 2016, **2**, e1600292.
27 B. Carpenter, R. Nehmé, T. Warne, A. G. Leslie and C. G. Tate, *Nature*, 2016, **536**, 104–107.
28 E. Segala, D. Guo, R. K. Cheng, A. Bortolato, F. Deflorian, A. S. Doré, J. C. Errey, L. H. Heitman, A. P. IJzerman, F. H. Marshall, and others, *Journal of medicinal chemistry*, 2016, **59**, 6470–6479.
29 G. Lebon, P. C. Edwards, A. G. Leslie and C. G. Tate, *Molecular pharmacology*, 2015, **87**, 907–915.
30 W. Liu, E. Chun, A. A. Thompson, P. Chubukov, F. Xu, V. Katritch, G. W. Han, C. B. Roth, L. H. Heitman, A. P. IJzerman, V. Cherezov and R. C. Stevens, *Science*, 2012, **337**, 232–236.
31 M. Congreve, S. P. Andrews, A. S. Doré, K. Hollenstein, E. Hurrell, C. J. Langmead, J. S. Mason, I. W. Ng, B. Tehan, A. Zhukov, and others, *Journal of medicinal chemistry*, 2012, **55**, 1898–1903.
32 T. Hino, T. Arakawa, H. Iwanari, T. Yurugi-Kobayashi, C. Ikeda-Suno, Y. Nakada-Nakura, O. Kusano-Arai, S. Weyand, T. Shimamura, N. Nomura, and others, *Nature*, 2012, **482**, 237–240.
33 A. S. Doré, N. Robertson, J. C. Errey, I. Ng, K. Hollenstein, B. Tehan, E. Hurrell, K. Bennett, M. Congreve, F. Magnani, and others, *Structure*, 2011, **19**, 1283–1293.
34 G. Lebon, T. Warne, P. C. Edwards, K. Bennett, C. J. Langmead, A. G. Leslie and C. G. Tate, *Nature*, 2011, **474**, 521–525.
35 F. Xu, H. Wu, V. Katritch, G. Han, K. Jacobson, Z. Gao, V. Cherezov and R. Stevens, *Science*, 2011, **332**, 322–327.
36 V.-P. Jaakola, M. T. Griffith, M. A. Hanson, V. Cherezov, E. Y. Chien, J. R. Lane, A. P. Ijzerman and R. C. Stevens, *Science*, 2008, **322**, 1211–1217.
37 J. Jiménez, M. Škalič, G. Martíez-Rosell and G. De Fabritiis, *J. Chem. Inf. Model.*, 2018, **58**, 287–296.
38 A. Gaulton, A. Hersey, M. Nowotka, A. P. Bento, J. Chambers, D. Mendez, P. Mutowo, F. Atkinson, L. J. Bellis, E. Cibrián-Uhalte, M. Davies, N. Dedman, A. Karlsson, M. P. Magariños, J. P. Overington, G. Papadatos, I. Smit and A. R. Leach, *Nucleic Acids Research*, 2016, **45**, D945–D954.
